# CRISPR/dCas9-KRAB-Mediated Suppression of *S100b* Restores p53-Mediated Apoptosis in Melanoma Cells

**DOI:** 10.3390/cells12050730

**Published:** 2023-02-24

**Authors:** Samrat Roy Choudhury, Billie Heflin, Erin Taylor, Brian Koss, Nathan L. Avaritt, Alan J. Tackett

**Affiliations:** 1 Pediatric Hematology-Oncology, Arkansas Children’s Research Institute, University of Arkansas for Medical Sciences, Little Rock, AR 72202, USA; 2 Department of Biochemistry & Molecular Biology, University of Arkansas for Medical Sciences, Little Rock, AR 72205, USA

**Keywords:** melanoma, *S100b*, CRISPR, dCas9-KRAB, apoptosis, cell death

## Abstract

Overexpression of S100B is routinely used for disease-staging and for determining prognostic outcomes in patients with malignant melanoma. Intracellular interactions between S100B and wild-type (WT)-p53 have been demonstrated to limit the availability of free WT-p53 in tumor cells, inhibiting the apoptotic signaling cascade. Herein, we demonstrate that, while oncogenic overexpression of *S100B* is poorly correlated (R < 0.3; *p* > 0.05) to alterations in *S100B* copy number or DNA methylation in primary patient samples, the transcriptional start site and upstream promoter of the gene are epigenetically primed in melanoma cells with predicted enrichment of activating transcription factors. Considering the regulatory role of activating transcription factors in *S100B* upregulation in melanoma, we stably suppressed *S100b* (murine ortholog) by using a catalytically inactive Cas9 (dCas9) fused to a transcriptional repressor, Krüppel-associated box (KRAB). Selective combination of *S100b*-specific single-guide RNAs and the dCas9-KRAB fusion significantly suppressed expression of *S100b* in murine B16 melanoma cells without noticeable off-target effects. *S100b* suppression resulted in recovery of intracellular WT-p53 and p21 levels and concomitant induction of apoptotic signaling. Expression levels of apoptogenic factors (i.e., apoptosis-inducing factor, caspase-3, and poly-ADP ribose polymerase) were altered in response to *S100b* suppression. *S100b*-suppressed cells also showed reduced cell viability and increased susceptibility to the chemotherapeutic agents, cisplatin and tunicamycin. Targeted suppression of *S100b* therefore offers a therapeutic vulnerability to overcome drug resistance in melanoma.

## 1. Introduction

Melanoma is one of the deadliest forms of skin cancer, is particularly prevalent (2.5%) among the Caucasian population, and has almost doubled in incidence over the past three decades in the United States alone [1]. Several serological biomarkers have been identified for early detection, staging, prognosis, and therapeutic determination of melanoma. Among them, a high serum level of S100B (S100 calcium-binding protein B) has emerged as the most reliable biomarker of progression and survival outcome of the disease [2,3]. 

S100B (10.7 kDa) is a member of the S100 protein family that binds to its molecular targets by undergoing conformational changes at the carboxy-terminal EF-hand motif. The affinity of S100B-mediated protein–protein interactions is strengthened with the increase in intracellular Ca^2+^ concentrations. Ectopic upregulation of the protein has been noted in metastatic melanomas, as well as proneuronal, neural, or classic types of gliomas [3,4,5]. In melanoma, intracellular S100B interacts with proteins of different signaling pathways [6], such that S100B activates the glycolytic enzyme fructose 1,6-biphosphate (aldolase) and increases metabolism of melanoma cells. The protein also interacts with cytoskeletal components such as tubulin, Rac1 (GTPase), or IQGAP1 (cdc42 effector), which alters motility of melanoma cells toward enhanced migration and invasion [7]. In contrast, when extracellularly secreted via the receptor for advanced glycation end products (RAGE) signal transduction pathway, S100B facilitates tumor development in a mouse model of melanoma [8]. Additionally, S100B triggers melanoma tumor growth by interacting with the C terminus of wild-type (WT)-p53, preventing protein tetramerization and covalent modifications (e.g., phosphorylation or ubiquitination) [9]. Therefore, S100B–p53 interaction lowers the intracellular availability of free WT-p53 and limits the tumor-suppressing function of *TP53* [6,10,11]. Previously, siRNA-mediated cell-type-specific targeted inhibition of the S100B–p53 complex was shown to rescue protein levels of WT-p53, phosphorylated p53, and downstream p21 [10]. The targeted inhibition also evoked poly-ADP ribose polymerase (PARP)-mediated apoptosis involving activation of caspase-3 or caspase-8 or aggregation of Fas death receptor upon ultraviolet irradiation [10]. Several attempts have been made to develop small-molecule inhibitors (e.g., pentamidine) that inhibit molecular interactions between the Ca^2+^-S100B apoprotein and its binding proteins [12]; however, the small-molecule inhibitors, as well as siRNA-mediated knockdown, of *S100B* resulted in only transient restriction of melanoma cell growth. 

We aimed to develop a more stable and effective targeted approach that uses a CRISPR (clustered regularly interspaced short palindromic repeats) platform for stable suppression of S100B protein. Targeted epigenome editing previously was attempted by using a CRISPR and associated protein 9 (Cas9) endonuclease system that contained deactivated Cas9 (dCas9) fused to a transcriptional suppressor, Krüppel-associated box repressor (KRAB) [13]. The KRAB domain exerts widespread transcriptional suppression through enrichment of H3K9me3 and condensation of chromatin [14]. Herein, we report development of a dCas9-KRAB (DK) system for targeted perturbation of *S100B* expression to rescue WT-p53 and its associated tumor-suppression properties in melanoma cells. 

## 2. Materials and Methods

### 2.1. TCGA Patient Cohorts

Data from 28 retrospective studies available through the Cancer Genome Atlas (TCGA) were used to examine *S100B* expression in samples from patients with cutaneous (*n* = 443) or ocular (*n* = 80) melanoma, as well as patients with different cancer types (*n* = 10,071; comparison group) (**Table S1**). From the TCGA Firehose Legacy study, we selected a cohort of patients with skin melanoma (*n* = 287) on the basis of available data for DNA methylation, copy number (CN) alterations, and gene expression from the matched patient samples (**Table S2**) [15]. Genetic and transcriptomic data from TCGA were extracted through the cBioPortal (www.cbioportal.org; accessed on 17 January 2022). GEP was analyzed from mRNA expression (RNA sequencing [RNA-seq]) data; we represent GEP data expressed in RNA-seq by expectation-maximization (RSEM), log2 scale. CN alterations are presented as the capped relative linear copy-number values, where we consider patients with diploid genomes for *S100B*, compared to those with gain (+1) or loss (–1) in CN. There were single cases for amplification (+2) or deep deletion (–2); these were excluded from the analysis. DNA methylation was assayed on an Illumina HumanMethylation450 (HM450) BeadChip platform (San Diego, CA, USA), and the average β-value from all probe sets against *S100B* was considered. Raw values for GEP, CN, and methylation from matched patients are summarized in **Table S3**. 

### 2.2. Proteomics Data Set from Cases of Melanoma Treated with Immune Checkpoint Inhibitors (ICIs)

A proteomics data set was analyzed from the consented (study #204543) and deidentified tissue biopsies from a cohort of patients with melanoma (*n* = 23) that was diagnosed at stage IV and treated with first-line ICIs (i.e., anti-CTLA4, PD-1, or a combination of monotherapies) at the University of Arkansas for Medical Sciences. The proteomics sample was prepared, and data were analyzed as reported earlier [16]. Clinicopathological information of the patients, including response to ICIs, is summarized in **Table S4**.

### 2.3. Identification of DNase Hypersensitive Sites (DHSs)

The chromatin state at the S100B promoter, including transcriptional start sites (TSSs) and intragenic regions, was examined by comparing DNase-sequencing data from SK-MEL-5 melanoma cell lines (accession ID: ENCFF627WEJ) to that from primary keratinocytes derived of newborn foreskin (accession ID: ENCFF910KFI); both data sets were acquired from the ENCODE database. The DHS signals were represented as read-depth normalized signal. S100B mRNA levels in the same cell line (accession ID: ENCFF249LKO) and primary keratinocytes (accession ID: ENCFF249BFY) were also analyzed. The bigwig (hg38) files for both DHSs and RNA-seq data were loaded on the integrative genomics viewer (v 2.14.0, Broad Institute, Cambridge, MA, USA) for visualization. 

### 2.4. Prediction of Homology between Human and Murine Proteins

The amino acid sequences of human (*S100B*) and murine (*S100b*) homologs of the protein were compared by using Clustal Omega (v 1.2.4) (EMBL-EBI, Hinxton, UK) function and were presented by using Jalview (v 2.11.2.0) [17]. 

### 2.5. Prediction of Transcription Factor (TF) Binding

The putative TFs that interact with the murine single-guide RNA (m-sgRNA)-binding sites on *S100b* were predicted by using the target regions as input for the PROMO database (http://alggen.lsi.upc.es/; accessed on 21 May 2020) that uses TRASFAC for the prediction analyses [18,19]. The output was specific to *Mus musculus.*

### 2.6. Development of CRISPR/dCas9-KRAB Tool

A plasmid containing the KRAB domain fused to dCas9 and the programmable sgRNA vector were obtained from Addgene, Watertown, MA, USA (#99372, and #44248, respectively). The plasmid #44248 contains an EGFP (enhancer green fluorescent protein)-specific sgRNA and has been used as an off-target control for the present study. Three murine *S100b*-specific sgRNAs were selected for the present study, based on their predicted efficacy of inhibition at the target site. m-sgRNA-1 and m-SgRNA-2 were selected from the sgRNA library that was recommended for the mouse genome [20]; m-SgRNA-3 was custom designed by using the CHOPCHOP (https://chopchop.cbu.uib.no/; accessed on 2 July 2020) algorithm design tool (**Table S5**). All sgRNA-binding sites were mapped with Mouse Genome Informatics (http://www.informatics.jax.org/; accessed on 12 June 2020) and annotated against the GRCm38/mm10 genome. The selected sgRNAs in combination with the scaffolds were obtained as gene blocks (gblocks) from Integrated DNA Technologies, Coralville, IA, USA. The gblocks were PCR amplified (**Table S6**) with CloneAmpHiFi PCR Premix (Takara, Kusatsu, Shiga, Japan) and were integrated into the sgRNA plasmid, flanked by BstXI and XhoI (New England Biolabs, Ipswich, MA, USA) sites. Correct clones were confirmed with low-throughput sequencing (**Table S6**, sequencing primers).

### 2.7. Cell Lines and Transduction

Murine B16-F1 (CRL-6323) melanoma and B16-F10 (CRL-6475) metastatic melanoma cells were purchased from ATCC (American Type Culture Collection, Manassas, VA, USA). Cell lines were cultured in Dulbecco’s Modified Eagle Medium supplemented with 10% FBS, 1% penicillin–streptomycin (Thermo–Fisher, Waltham, MA, USA), and 1% glutamine and maintained at 37 °C and 5% CO_2_. Cells were transduced with plasmid encoding DK alone, or in combination with the plasmids encoding sgRNAs specific to *S100b* or *EGFP* (sgRNA^EGFP^). Transduction was accomplished with a one-step lentivirus packaging system (Takara, Kusatsu, Shiga, Japan) per the manufacturer’s instructions. Briefly, 4–5 × 10^6^ LentiX-293T cells (Takara) were transfected with the plasmids in Opti-MEM reduced serum medium (Thermo–Fisher, Waltham, MA, USA) and were incubated for 4–6 h. Next, each culture was supplemented with 14 mL complete medium and then incubated for 48 h. Supernatants were collected, passed through 0.45 µM syringe filters, and added dropwise to polybrene (8 µg/mL) charged F1 or F10 cells. Cells co-transduced with DK and sgRNA constructs were selected with puromycin (1 µg/mL), followed by FACS sorting (FACSARIA III, BD, San Jose, CA, USA) for mCherry^+^ cells (>95%). 

### 2.8. Quantitation of Expression with Quantitative Real-Time PCR (qPCR)

Expression of target genes was determined in reference to endogenous *GAPDH*, out of total RNA extracted from the cells. From cells that had been treated for 24 h, total RNA was extracted (RNeasy Mini Kit, QIAGEN, Hilden, Germany) and converted to c-DNA (Iscript Advanced cDNA synthesis kit, Bio-Rad, Hercules, CA, USA). The fold change in *S100b* expression was then determined, in triplicate, with qPCR (StepOnePlus Real-Time PCR Systems; v 2.0 Applied Biosystems, Waltham, MA, USA), compatible with SYBR green master mix (Thermo–Fisher). Amplification reactions were carried out at 95 °C for 1 min, followed by 40 cycles at 95 °C for 15 s and 60 °C for 1 min. Primers used to amplify murine *S100b* (qMmuCID0015305), *TP53* (qMmuCIP0032520), and *GAPDH* (qMmuCED0027497) were purchased from commercial sources (PrimePCR SYBR Green Assay, Bio-Rad, Hercules, CA, USA). The primers for *DIP2A*, *PRMT2*, *CDKN1A, AIFM1, CASP3, PARP-1*, and *GAPDH* were custom synthesized with IDT (Integrated DNA Technologies, Coralville, IA, USA), and primer sequences are summarized in **Table S7**. 

### 2.9. Western Blot Analysis of Protein Levels

We prepared whole-cell lysates by using RIPA buffer (Thermo–Fisher, Waltham, MA, USA) per the manufacturer’s instructions. To determine levels of S100b protein, an aliquot of lysate (40 µg total protein, determined with the BCA assay; Pierce) was loaded onto 4–20% Bis–Tris gels (Thermo–Fisher, Waltham, MA, USA); to determine levels of p21, p53, AIFM1, cleaved Caspase-3, or PARP-1 proteins, aliquots of lysate (approximately 20 µg total protein) were loaded onto 4–12% Bis-Tris gels (Thermo–Fisher, Waltham, MA, USA), and proteins were electrotransferred to PVDF membranes (Bio-Rad, Hercules, CA, USA). The blots were blocked in 5% milk in TBST for 1 h, followed by overnight incubation at 4 °C with primary antibodies in 1% milk. Blots were probed with primary antibodies (**Table S8**) per the manufacturer’s instructions. Blots were then washed 3 times with 1% milk and incubated with HRP-tagged secondary antibody for 1 hour. Finally, blots were washed 3 times in TBST and were developed with enhanced chemiluminescence reagents (Perkin Elmer, Waltham, MA). Densitometries of the bands from the blots (wherever applicable) were prepared with ImageJ (https://imagej.nih.gov/ij/; accessed on 23 February 2023), v 1.53t, National Institute of Health, Bethesda, MD, USA). 

### 2.10. Apoptosis and Cell Death Assay

F1 and F10 cells were seeded in 6-well plates (5 × 10^4^ cells per well). After 24 h, they were treated with staurosporine (Seleckchem, Houston, TX, USA) at indicated doses for 24 h. Apoptosis and cellular mortality then were determined with flow cytometry (FACS Verse, BD Biosciences, San Jose, CA, USA); staining with annexin V-FITC (Biolegend, San Diego, CA, USA) and DAPI (Sigma, St. Louis, MO, USA) was used to determine the percent viable (i.e., Annexin V–, DAPI–) cells. 

### 2.11. Cell Proliferation and Chemotherapeutic Sensitivity Assay

Both B16 melanoma cell lines were seeded (5 × 10^4^ cells per well) in 12-well plates and imaged (from at least 10 different fields) after 24 and 48 h to examine differences in cell proliferation between the treatment and control groups. Additionally, B16 melanoma cells were seeded in 96-well plates (5 × 10^3^ cells per well), incubated for 24 h, and treated as indicated. Viability of co-transduced (DK + m-sgRNA-1) cells and untreated control cells was determined with a CellTiter-Glo Luminescent Cell Viability Assay kit (G7570, Promega, Madison, WI, USA) after 24 and 48 h in the presence or absence of two chemotherapeutic agents, cisplatin (13119, Cayman Chemicals, Ann Arbor, MI, USA) and tunicamycin (11445, Cayman Chemicals, Ann Arbor, MI, USA). Drugs were serially diluted from the stock solution (2 mM) and added to cell cultures in the range of 5 µM to 10 nM. At the end of 24 and 48 h of incubation, equal volumes (100 µL each) of assay solution and cell suspension were mixed and incubated at room temperature for 10 min. Relative luminescence was recorded with a spectrometer (Agilent BioTek, Winooski, VT, USA Gen5 Microplate reader), and the half-maximal inhibitory concentration (IC_50_) value for each drug was plotted with GraphPad Prism, v7.0, Boston, MA, USA.

### 2.12. Statistical Analysis

We used the non-parametric Mann–Whitney U test or Dunn’s multiple comparison test to determine the significance of differences between the groups being compared. Significance was defined as *p* < 0.05. Statistical analyses and associated graphs were generated with GraphPad Prism, Boston, MA, USA.

## 3. Results 

### 3.1. Overexpression of S100B Is Linked to Copy-Number Changes and Epigenetic Alterations of the Gene 

**Figure 1:**
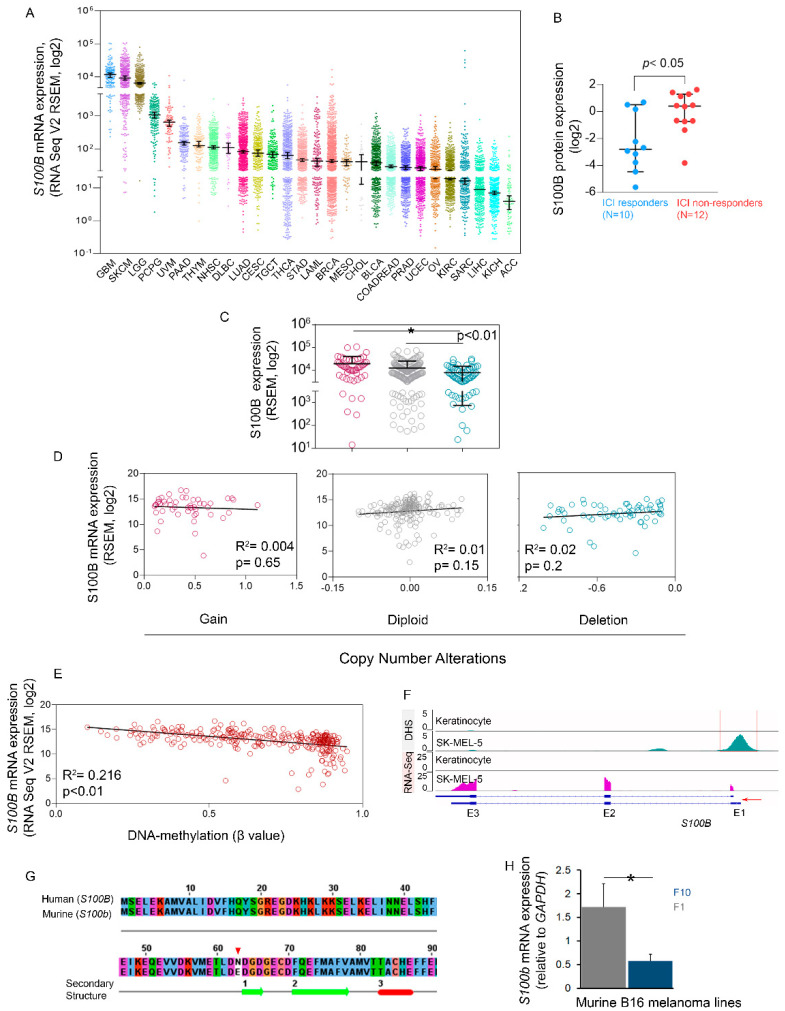
(**A**) Median mRNA expression (RNA-seq V2, RSEM, log2) of *S100B* in patients from 28 retrospective studies, available from the Broad GDAC Firehose database. S100B expression was measured in GBM (glioblastoma; *n* = 160), SKCM (skin cutaneous melanoma; *n* = 443), LGG (glioma; *n* = 514), PCPG (pheochromocytoma; *n* = 178), UVM (ocular melanoma; *n* = 80), PAAD (pancreatic cancer; *n* = 177), THYM (thymic epithelial tumor; *n* = 119), HNSC (head and neck cancer; *n* = 515), DLBC (mature B-cell neoplasms; *n* = 48), LUAD (non-small cell lung carcinoma; *n* = 994), CESC (cervical cancer; *n* = 294), TGCT (seminoma; *n* = 149), THCA (thyroid cancer; *n* = 498), STAD (esophagogastric cancer; *n* = 593), LAML (leukemia; *n* = 173), BRCA (breast cancer; *n* = 1082), MESO (pleural mesothelioma; *n* = 87), CHOL (cholangiocarcinoma; *n* = 36), BLCA (bladder cancer; *n* = 407), COADREAD (colorectal cancer; *n* = 592), PRAD (prostate cancer; *n* = 493), UCEC (endometrial cancer; *n* = 584), OV (ovarian epithelial tumor; *n* = 300), KIRC (renal clear cell carcinoma; *n* = 510), SARC (sarcoma; *n* = 253), LIHC (hepatobiliary cancer; *n* = 366), KICH (renal non-clear cell carcinoma; *n* = 348), ACC (adrenocortical carcinoma; *n* = 78). (**B**) S100B protein expression in a cohort of SKCM patients non-responsive (*n* = 12) to immune-checkpoint inhibitors (ICI; anti-PD-1 monotherapy, anti-CTLA-4 monotherapy, and anti-PD-1/anti-CTLA-4 combination therapy), compared to the responsive cohort (*n* = 10). (**C**) *S100B* expression in SKCM patient samples with gains in copy number, compared to those with heterozygous deletion of the gene (*p* < 0.01). (**D**) Correlation between linear capped copy number values and mRNA levels of *S100B* in SKCM samples. (**E**) A moderately negative correlation (R^2^ = 0.216, *p* < 0.01) was observed between DNA methylation (β-values) and mRNA expression of *S100B*. (**F**) DNase hypersensitive sites (DHSs; determined with DNase sequencing) and RNA-signal intensities (determined with RNA sequencing [RNA-seq]) in melanoma (SK-MEL-5) and in primary keratinocytes. (**G**) The amino acid sequence of human S100B and its murine ortholog S100b shows the degree of conservation between the species, with a single amino acid substituted (red arrowhead). (**H**) mRNA levels of *S100b* (relative to those of *GAPDH*) in murine B16-F1 (blue) and B16-F10 (gray) melanoma cells. * *p* < 0.05.

We evaluated *S100B* expression (RNA-seq V2, RSEM, log2) in 10,071 patient-derived samples of 28 different cancers from the TCGA repository (**Table S1**). Expression of *S100B* was highest in glioblastoma, followed by skin cutaneous melanoma (SKCM). *S100B* expression in SKCM samples (*n* = 443) ranged (25–75% quartile) from 4287.6 to 16,048.4 with a median of 8959.4 ± 14,212.4 (standard deviation). In samples of ocular melanoma (UVM) (*n* = 80), the gene was expressed at a similar fashion to SKCM or was overexpressed (646.7 ± 1474.9), relative to other cancer types (**Figure 1A**). A separate in-house proteomics study of a cohort of patient-derived SKCM samples demonstrated that, in the subgroup (*n* = 12) that was non-responsive to ICIs anti-CTLA-4, anti-PD1, and anti-CTLA4 + anti-PD1 combined therapy, the S100B protein was upregulated (*p* < 0.05, >2-fold change), compared to the subgroup (*n* = 10) that was responsive (**Figure 1B**). These findings strongly suggest that ectopic upregulation of *S100B* might play a critical role in development of refractory SKCM [10,21] and could contribute to therapeutic resistance in the disease. 

Next, we combined the data on *S100B* expression with data on CN alterations and DNA-methylation profiles in matched SKCM samples (*n* = 285), available from TCGA. We observed that 17.5% (50/285) of samples had gains in *S100B* CN, and 21% (60/285) of samples had heterozygous deletions in CN of *S100B*, while 61.4% (175/285) of samples were diploid for *S100B*. A significant (*p* < 0.01) decrease in median expression of *S100B* (RSEM = 5624.3) was observed in samples with CN losses, compared to those with gains (RSEM = 14,119.5) (**Figure 1C**) but not to those with diploidy. However, weak correlations (*p* > 0.05; R^2^ < 0.2) were observed when the relative linear values of CN for individual subgroups (gain, loss, or diploid) and their expression profiles were compared (**Figure 1D**). We also investigated whether *S100B* expression correlates to DNA methylation (β-values, probe: cg12092309) of *S100B*. Methylation ranged (25–75% quartile) from 0.54 to 0.87, with a median value 0.74. We observed a moderate (R^2^ = 0.216; *p* < 0.01) negative correlation between *S100B* methylation level and expression in SKCM samples, such that samples with lower methylation levels have relatively higher expression and vice versa (**Figure 1E**). Additionally, we analyzed signal intensities based onDHS (DNAse hypersensitive sites) to assess the chromatin state of *S100B* in the SK-MEL-5 cell line, which is representative of human SKCM, and compared it to that in primary keratinocytes derived from foreskin of human newborns. We observed epigenetic priming in the melanoma cells, such that DHS enrichment was observed in SK-MEL-5 cells at the *S100B* TSS and upstream promoter, which spans 578 bp (chr21:46,604,961-46,605,537) (**Figure 1F**). The conformational change in chromatin state may be best linked to *S100B* upregulation in melanoma. This is supported by RNA-seq analysis of the same set of samples, which revealed no *S100B* transcript in primary keratinocytes but marked *S100B* transcript from exon (E)2 and E3 in SK-MEL-5.

Next, we evaluated amino acid sequence homology between human S100B and its mouse ortholog S100b. The analysis showed that the proteins are almost identical. Each contains 92 amino acids with a single base substitution (asparagine to glutamic acid) at position 63 (**Figure 1G**), which is predicted not to significantly alter the protein’s secondary structure (Jalview 2.11.1.0). Mouse B16 melanoma cells have been used for expressing differential levels of S100b under different pathological conditions in conjunction with tumor development [22,23,24]. We used the F1 and metastatic F10 melanoma cell lines to determine whether suppression of the gene can evoke anticancer phenotypes in treated cells. *S100b* expression (signal intensity relative to endogenous *GAPDH*) in F10 cells (0.58) was significantly (*p* < 0.05) lower than in F1 cells (1.72) (**Figure 1H**).

### 3.2. S100b Expression Is Suppressed by CRISPR/dCAS9-KRAB

**Figure 2:**
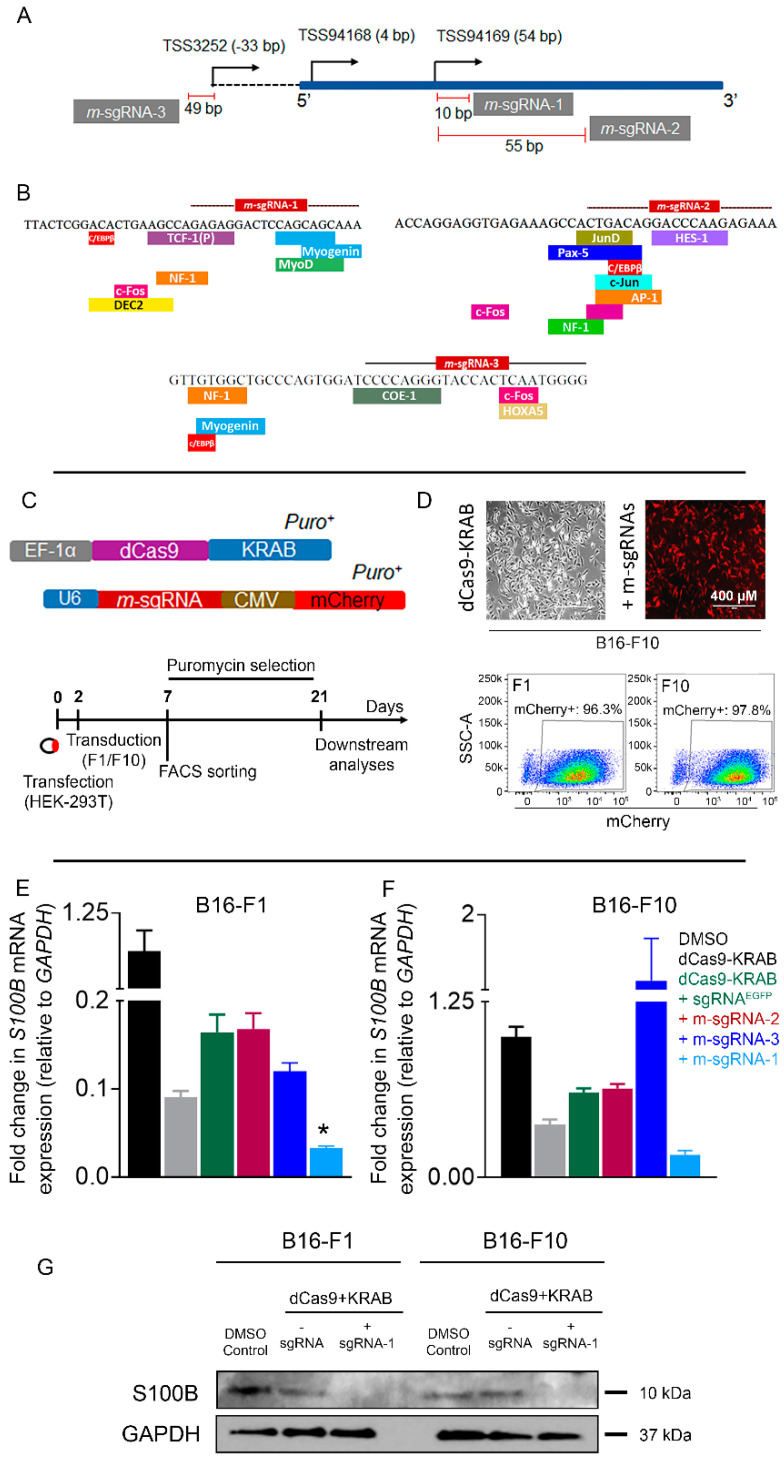
(**A**) Murine single-guide RNAs (m-sgRNAs) were designed to bind proximal sites of three possible transcription start sites (TSSs) of *S100b.* (**B**) Prediction of transcription factor (TF) binding at the m-sgRNA-1 (+10 bp of the TSS94169), m-sgRNA-2 (+55 bp of the TSS94169), and m-sgRNA-3 (–33 bp of the TSS3252) target sites were mapped per Mouse Genome Informatics. (**C**) Schematics representing the fusion of dCas9 and KRAB proteins, *S100b*-specific m-sgRNAs, and plan for selecting transduced cells prior to downstream experiments. (**D**) Representative bright-field (dCas9-KRAB) and fluorescence (dCas9-KRAB+m-sgRNAs) micrographs (400 µM scale) of B16-F10 cells (above); fluorescence-assisted cell sorting (FACS) demonstrating enrichment (>95%) of co-transduced B16-F1 or B16-F10 cells. (**E**,**F**) Fold change in *S100b* expression (normalized to *GADPH*) in B16-F1 melanoma cell line (**E**) and B16-F10 melanoma cell line (**F**). Control (parental cell line, not transduced; black, treated with 0.1% dimethyl sulfoxide)); transduced with dCas9-KRAB only (light gray), with dCas9-KRAB+sgRNA^EGFP^ (green), dCas9-KRAB+m-sgRNA-2 (red), with dCas9-KRAB+m-sgRNA-3 (dark blue), or with dCas9-KRAB+m-sgRNA-1 (light blue); * *p* < 0.05. (**G**) Western blot images for S100b expression in F1 and F10 cells, treated with the combination of dCas9-KRAB plus sgRNA-1, compared to cells treated with dCas9-KRAB alone and untreated cells.

We aimed to suppress the *S100b* expression by using CRISPR-KRAB-mediated interference at selected loci of the gene. *S100b*-specific m-sgRNAs were selected from the previously reported CRISPR inhibition library specific for the mouse genome or were designed with an online algorithm tool [20]. Ref. [25] m-SgRNA-1 and m-SgRNA-2 were targeted at 10 bp and 55 bp, respectively, downstream from the *S100b* TSS (chr10:76253899-76253915); m-SgRNA-3 was targeted at 49 bp upstream of the TSS (chr10:76253817-76253822) (**Figure 2A**). The targeted *S100b* regions also contain putative binding sites (predicted by the TRANSFAC tool) for multiple TFs. In particular, we observed the prevalence of c-Fos, C/EBP-β, and NF-1 TFs within 20 bp of the target sites of the 3 m-sgRNAs, suggesting their putative regulatory roles in determining upregulation of *S100b* (**Figure 2B**) [26,27,28]. The DK repressor protein was stably expressed in F1 and F10 cells via lentiviral transduction and was maintained under puromycin selection (**Figure 2C**). Additionally, the m-sgRNA vectors harboring an mCherry fluorophore allowed the use of FACS sorting to enrich (>95%) co-transduced cells (**Figure 2D**). The cells were transduced with DK module with or without three individual *S100B*-specific sgRNAs. Cells transduced with DK in combination with sgRNA^EGFP^ served as an off-target control for the current study. 

The inherent transcription-repressive nature of the KRAB protein resulted in reduced *S100b* mRNA levels in both F1 and F10 cell lines, whereas combinatorial treatment with DK plus selective m-sgRNAs resulted in different degrees of reduction in the gene expression, compared to the parental lines (except for m-sgRNA-3 in F10). Therefore, to control for the effects of KRAB, we used DK-transduced cells as the baseline to which we compared the cells that were also transduced with the m-sgRNAs, for determining their effects on *S100b* expression. *S100b* expression was significantly (*p* < 0.05) reduced in the B16-F1 cells treated with the combination of DK plus m-sg-RNA-1, compared to the cells treated with DK alone (**Figure 2E**). In contrast, *S100b* expression was increased in F1 cells when treated with the combinations of DK plus remaining *S100b*-specific sgRNAs (m-sgRNA-2/3) or sgRNA^EGFP^ (**Figure 2E**). A similar pattern was observed in the change in *S100b* mRNA expression in the B16-F10 cells, such that the gene expression was further reduced (*p* > 0.05) in cells treated with the combination of DK and m-sg-RNA-1, compared to cells transduced with DK alone. 

The combination of DK with m-SgRNA-2/3 or with sgRNA^EGFP^ in either type of B16 cells remained ineffective in suppressing *S100b* expression. Additionally, *S100b* expression remained unaltered or insignificantly changed in both cell lines when transduced with vectors containing individual m-sgRNAs specific to *S100b* or *EGFP* without the KRAB module, compared to non-transduced parental lines (**Figure S1**). We observed a significant decrease in S100b mRNA expression in both the B16 cells, transduced with DK fusion alone, whereas the protein expression completely disappeared in cells transduced with the combination of DK plus m-SgRNA-1. Therefore, the S100b protein-expression changes in the similar direction to the changes in mRNA level in both F1 and F10 cells. Western blot results also corroborated the fact that S100b is much less expressed, both at the level of mRNA (**Figure 1H**) and protein (**Figure 2G**) in the F10 cells compared to the F1 cells. However, the S100b protein-expression band was totally resolved in the F10 cells treated in combination with DK plus m-SgRNA-1. 

**Figure 3:**
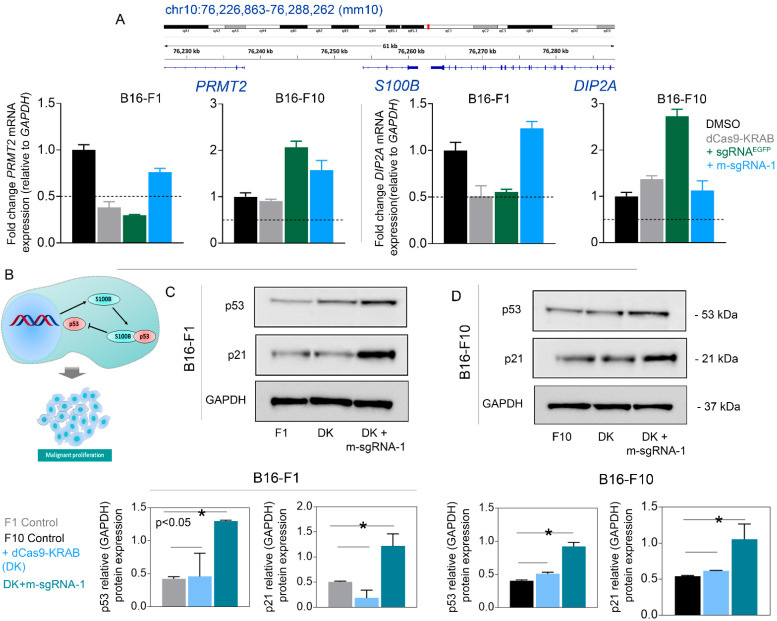
(**A**) Genomic coordinates of *S100B* and its neighboring genes *PRMT2* and *DIP2A.* The mRNA-expression of both genes was monitored with qPCR, followed by the CRISPR-intervention with the combination of dCas9-KRAB and sgRNA, specific to *S100b* or *EGFP*. (**B**) Schematic representation of S100b interaction with wild type (WT)-p53 within the nucleus, potentially resulting in limited intracellular levels of p53 protein and inducing malignant cell proliferation in melanoma. (**C**,**D**) Western blot and associated densitometric plots demonstrating significant (* *p* < 0.05) increases in p53 and downstream p21 levels in B16-F1 and B16-F10 melanoma cell lines in response to co-transduction with dCas9-KRAB and m-sgRNA-1.

Next, to examine the specificity of the CRISPR-KRAB toolbox developed here, we looked into the possible changes in expression of *PRMT2* and *DIP2A*, two adjacent genes to *S100b* on Chr.10 (mm10) (**Figure 3A**). We did not observe any significant (*p* > 0.05) reduction in the expression of *PRMT2* and *DIP2A*, indicating that the action of CRISPR-KRAB interference was target-specific (**Figure 3A**). In summary, the combination of DK plus m-SgRNA-1 imparted the greatest repressive effects on *S100b* expression, and CRISPR-KRAB interference was more profound in F1 cells than in F10 cells. Therefore, we continued with this combination for the downstream biological analyses.

### 3.3. S100b Suppression Restores WT-p53 Level and Activates Apoptotic Signaling

Based on the previous literature [2,10], we hypothesized that DK-mediated suppression of *S100b* might increase the availability of intracellular free WT-p53 and reactivate the tumor suppressor function of *TP53* (**Figure 3B**). DK plus m-SgRNA-1 resulted in no observed increases in mRNA levels of *TP53* in F1 or F10 cells (**Figure S2**). However, in both cell types, co-transduction with DK plus m-SgRNA-1 resulted in significantly increased (*p* < 0.05) p53 protein levels (**Figure 3C,D**), while transduction with only DK resulted in no marked changes in p53 protein levels in both cell types. As previously mentioned, targeted inhibition of *S100b* may release intracellular S100b-bound p53, potentially increasing levels of WT-p53 and its downstream product p21 or apoptogenic proteins [10,29,30]. Therefore, to investigate the consequences of *S100b* suppression, we assessed the effects of DK on induction of apoptosis in the target cells. In both F1 and F10 cells, transduction with DK plus m-SgRNA-1 resulted in significantly increased (*p* < 0.05) expression of *CDKN1A* (p21) in F1 cells both at the level of mRNA (14% increase) (**Figure S2**) and protein (**Figure 3C**), compared to F1-control. In contrast, a marginal (*p* = 0.05) increase in *CDKN1A* (11%) expression was observed in F10 cells (**Figure S2**). Nonetheless, the p21 protein level was significantly increased (*p* < 0.05) in the F10 line, compared to the control (**Figure 3D**).

**Figure 4:**
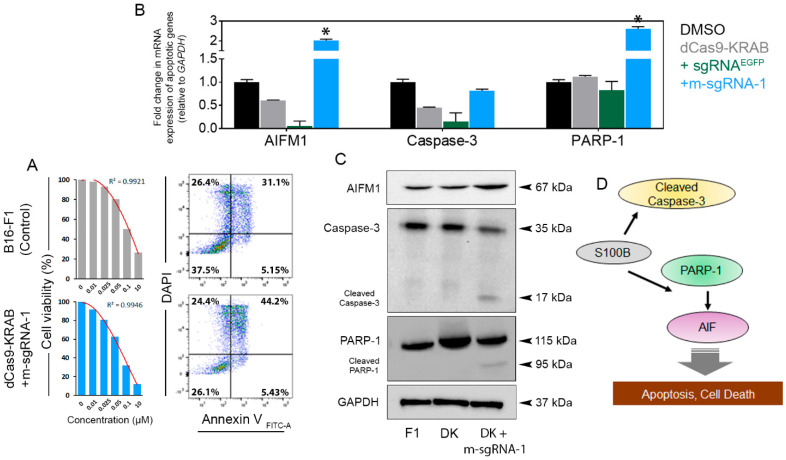
(**A**) Viability of B16-F1 cells (control: parental cells with no transduction) in response to increasing concentrations of staurosporine, a potent inductor of apoptosis. Representative images (0.1 µM) demonstrating enhanced cell death (44.2%) in the dCas9-KRAB+m-sgRNA-1 suppressed cells compared to the non-transduced controls (31.2%). (**B**) We observed significant (* *p* < 0.05) increase in *AIFM1* and *PARP-1* mRNA expression in F1 cells, transduced in combination with DK and m-sgRNA-1, compared to the cells treated with treated with 0.1% dimethyl sulfoxide (DMSO), DK alone or in combination with EGFP-specific sgRNA. However, no significant (*p* > 0.05) change was observed in the mRNA level of *CASP3* (Caspase-3) upon transduction with DK and different combinations of sgRNAs, compared to the parental line. (**C**) Changes in protein expression of apoptosis-responsive proteins, such that AIFM1 expression was increased, whereas we recognized cleaved forms of caspase-3 and poly-ADP ribose polymerase (PARP)-1 in the treated cells. (**D**) Schematic illustration of the potential apoptotic mechanism based on our findings that inhibition of S100b may increase the level of caspases or AIF1 to evoke apoptotic responses in cells.

To test the effects of elevated p53 and p21 levels on apoptosis, we treated the control (F1/F10) and DK-m-SgRNA-1 transduced cells with staurosporine (STS), a generic inducer of apoptosis. S100b-suppressed F1 cells, compared to the parental line, had increased cell death and susceptibility to serial concentrations (0.1 nM, 0.05 nM, 0.025 nM, 0.01 nM) of STS. An untreated control and exceedingly high dose (10 µM) of STS were used as negative and positive controls for cell viability assays. For instance, at 0.1 µM STS, we observed 11% less cell viability in F1 cells transduced with DK plus m-SgRNA-1 (26.1%) than in the parental cells (37.5%) (**Figure 4A**); however, we did not observe similar significant changes in cell death in studies with the F10 cells (data not shown). 

Next, to further investigate the observed cell death in the *S100b*-suppressed cells, we examined expression of key caspase and non-caspase proteases that are involved in apoptotic signaling pathways. We started by determining expression of mitochondrial apoptosis-inducing factor (AIFM1) [31,32], which was significantly increased (*p* < 0.05) in both mRNA and protein expression in the DK+m-SgRNA-1-transduced F1 cells, compared to non-transduced and DK+/-sgRNA^EGFP^ control cells (**Figure 4B,C**). The impact of differential upregulation of *AIFM1* then was evaluated in conjunction with caspase-3 activation.

We did not observe any significant changes in the *CASP3* mRNA expression in DK+m-SgRNA-1 transduced cells (**Figure 4B**). In contrast, a capase-3 protein cleavage (~17 kDa) was evident in cells transduced with DK+m-SgRNA-1 (**Figure 4C**); however, we did not observe noticeable changes in caspase-8 mRNA or protein levels (data not shown). Finally, we examined whether DK-m-SgRNA-1 affects expression of PARP-1, the substrate of caspase-3 [33]. We detected a significant (*p* > 0.05) increase in *PARP*-1 mRNA level (**Figure 4B**), while the cleaved subunit (~95 kDa) of PARP-1 protein was observed in DK+m-sgRNA-1 transduced cells but not in control cells (**Figure 4C**). In contrast, F10 cells transduced with DK+/-m-sgRNA-1 or sgRNA^EGFP^ failed to evoke any apoptogenic changes, compared to the control cells. In F10 cells, *S100b* suppression also did not result in altered protein levels of caspase-3/caspase-8 or in cleaved PARP-1 (**Figure S3**), indicating that an apoptotic response was not evoked. In summary, DK suppression of *S100b* efficiently evoked p53-mediated mitochondrial apoptotic machinery in F1 melanoma cells (**Figure 4C**) but not in F10 cells, despite elevation of WT-p53 and p21 proteins in these cells. This suggests a mechanistic difference in S100b regulation between the B16 lines, which needs further investigation.

### 3.4. S100b Suppression in Melanoma Cells Decreases Their Viability and Increases Their Susceptibility to Chemotherapeutics

**Figure 5:**
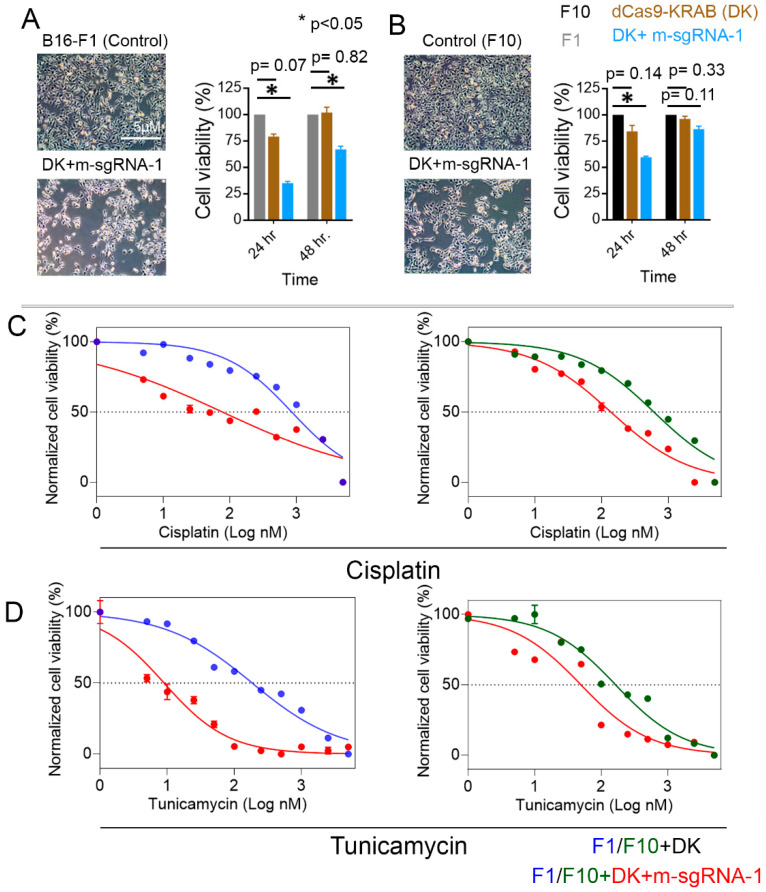
(**A**,**B**) Cell viability (%) was evaluated in B16-F1 and B16-F10 melanoma cell lines co-transduced with dCas9-KRAB and m-sgRNA-1, compared to control cells (no transduction), 24 h and 48 h after seeding the cells at a level of significance (* *p* < 0.05). (**C**,**D**) Viability of B16-F1 and B16-F10 melanoma cell lines in response to increasing concentrations of cisplatin or tunicamycin; control (parental cell line, transduced with dCas9+KRAB; F1: blue, F10: green); transduced with dCas9-KRAB plus sgRNA-1 (red).

We observed reduced cell viability in both the F1 and F10 cells co-transduced with DK in combination with m-SgRNA-1. We determined the percentage of viability by determining the amount of ATP produced by live cells 24 h and 48 h after initial seeding of cells in a 96-well plate (1 × 10^4^ cells per well). Compared to the control cells, co-transduced F1 cells had a 65% reduction and F10 cells had a 40% reduction (*p* < 0.05) in cell viability (**Figure 5A,B**) after 24 h. In contrast, after 48 h, F10 cells co-transduced with DK plus m-SgRNA-1 had only 13% less viability (*p* = 0.14) than control cells, but co-transduced F1 cells continued to have at least 32% less viability (*p* < 0.05) than control cells. 

Next, we treated *S100b*-suppressed F1 and F10 cells with chemotherapeutic agents against metastatic melanoma: cisplatin, a platinum analog, and tunicamycin, an inducer of endoplasmic reticulum stress [34,35]. We determined the IC_50_ after incubating cells with each drug for 24 h; as control comparison groups, we used F1 and F10 cells that were not CRISPR-transformed. For controlling melanoma cell growth, tunicamycin was found to be more efficacious than cisplatin. The IC_50_ against cisplatin was achieved at 77.5 nM (R^2^ = 0.83) in the *S100b*-suppressed F1 cells, compared to the F1 control cells (IC_50_ = 851.3 nM; R^2^ = 0.93); the IC_50_ against cisplatin was achieved at 138.4 nM (R^2^ = 0.97) in the *S100b*-suppressed F10 cells, compared to the F10 control cells (IC_50_ = 611.8 nM; R^2^ = 0.92) (**Figure 5C**). The IC_50_ against tunicamycin, however, was achieved at a concentration as low as 9 nM (R^2^ = 0.93) in the *S100b*-suppressed F1 cells, compared to 185.3 nM (R^2^ = 0.96) in the F1 control cells. In contrast, in the *S100b*-suppressed F10 cells, the IC_50_ of tunicamycin was attained at 50 nM (R^2^ = 0.91), compared to 165 nM (R^2^ = 0.96) in the F10 control cells (**Figure 5D**). Overall, our data suggest that *S100B* suppression cooperatively works with the chemotherapeutic agents that trigger cell death by evoking apoptotic responses, thus potentially sensitizing melanoma cells to these inhibitors.

### 3.5. Discussion

Overexpression of *S100B* and its clinicopathological relevance in melanoma has been well studied. The study reported here elucidated the role of genetic and epigenetic modifications underlying oncogenic overexpression of *S100B*. For instance, we demonstrated that *S100B* expression is significantly (*p* < 0.05) higher in patient-samples with gains in CN of the gene, compared to those with losses in CN of the gene (**Figure 1C**). However, this observation needs further validation and inclusion of more study cohorts, because we did not observe significant alterations in gene expression levels when comparing samples with losses in *S100B* CN and those diploid for *S100B*. At the epigenetic level, we investigated alterations in DNA methylation and chromatin state of the gene. We observed a moderate negative correlation between DNA methylation and expression, suggesting that the HM450 probe possibly is targeted to the promoter of the gene, where DNA methylation is inversely proportional to gene expression [36]. At the chromatin level, we observed an event of epigenetic priming at the *S100B* promoter, such that the TSS at the upstream promoter showed enrichment for DHS in SK-MEL-5 melanoma cells. This seems reliable because, in primary keratinocytes, the same region remains devoid of such DHS marks. The chromatin state alterations of *S100B* in melanoma cells could be logically ascribed to overexpression of the gene because it does not seem to be expressed in primary keratinocytes at levels comparable to those in melanoma cells. 

Enhanced molecular interactions between S100B and WT-p53 proteins in melanoma patient samples were demonstrated to impair WT-p53 function for tumor suppression through restricted cell cycle arrest [29], which leads to increased resistance to chemotherapeutics [11,12,37,38,39]. In the present study, we customized inactivation of *S100b* expression in two murine melanoma cell lines by using a CRISPR/dCas9 system that allowed for restoring intercellular levels of WT-p53 that might otherwise be bound to the S100b protein, thereby salvaging p53-mediated cell death and apoptosis. Our approach could be broadly applicable to most melanoma cases, irrespective of S100B CN gain or loss because the CRISPR/Cas9 system can intrinsically suppress multiple copies of the same gene [40]. 

We observed varying degrees of DK efficacy among the tested sgRNAs, specific to S100b or specific to *EGFP* and cell lines, which may be attributed to several factors. For instance, we observed little to no S100b-inhibition in either cell types transduced with a combination of DK plus m-SgRNA-2 or m-SgRNA-3. This could be partly due to the fact that the target region is already masked or occupied with endogenous TFs, leaving a relatively narrow window of DNA sequence available for binding sgRNAs or dCas9. We observed that the combination of m-SgRNA-1 and DK imparted the strongest suppression of *S100b* expression in both cell lines that were studied. However, combining DK with all three m-sgRNAs did not result in better suppression of *S100b* (data not shown), which could be partly related to the inefficacy of m-SgRNA-2 and m-SgRNA-3. For the current study, cells transduced with DK plus sgRNA^EGFP^ were used as an off-target control. It was observed that induction of cells with DK alone or DK in combination with *S100b*-specific sgRNA-2/3 or gRNA^EGFP^ reduced the gene expression, compared to the parental lines (**Figure 2E,F**), which, however, did not correlate to the changes in downstream apoptotic responses. Therefore, the generic reduction in *S100b* expression may be ascribed to the lentiviral effect on cells, which was not consistent with the downstream apoptotic signaling. In contrast, reduction of *S100b* expression was consistent with the expression of apoptosis-responsive proteins. This makes the combination of DK plus m-sgRNA-1 a unique and selective candidate for the intended purpose. 

*S100b* suppression significantly (*p* < 0.05) increased levels of p53 and its downstream p21 protein. However, CRISPR interference was not evident at the mRNA level for *TP53* in either cell line (**Figure S2**). This could be explained by the fact that *S100B* does not act upstream of the *TP53* signaling cascade and, therefore, may not have significant effects on expression of the gene. The disconnect between p53 mRNA and protein levels also aligned with results of a previous study in which siRNA-based perturbation of *S100b* did not alter *TP53* mRNA levels but significantly increased levels of total p53 and of phosphorylated p53 [10]. Overall, our findings support the hypothesis that suppressed S100b possibly lost its affinity for intracellular p53, thereby facilitating elevated levels of WT-p53 protein. We also observed that the degree of S100b inhibition correlated with p53-mediated activation and apoptotic changes, such that S100b inactivation and apoptotic changes in the F1 cells were profound, compared to those in the F10 cells. The decreased efficacy of DK against the F10 cells may be due to intrinsically lower expression of *S100b* in F10, relative to F1 cells, or due to ineffective binding of m-SgRNA-1 to the target region, presumably due to occupancy by other endogenous TFs. Disparities in the degree of functional outcomes between the two cell lines also could be explained by findings from previous studies reporting differential cytogenetic properties [41]. In this case, optimization of the KRAB suppressor system by integrating additional repressors, such as MeCP2 or EZH2, may impart superior gene inactivation effects against the F10 cells [42,43]. 

Finally, S100b inhibition decreased cell viability (**Figure 5A,B**) and increased susceptibility (**Figure 5C,D**) of melanoma cells to the chemotherapeutics. A previous study found cisplatin to be moderately effective against melanoma patients [35]. Similar findings were observed for the tunicamycin treatment. Similar to the S100b inhibition and apoptotic responses, chemotherapeutic susceptibility was also achieved at relatively lower concentrations in F1, compared to the F10 cells. 

A rational follow-up of the present study is aimed at determining the efficacy and persistence of the DK/CRISPR toolbox in other human and murine melanoma lines having high levels of S100b. Another limitation of the current study is that the S100b-specific DK/CRISPR tool needs further functional validations in vivo. Accordingly, the toolbox might need further modifications such that we may need to introduce additional suppressor domains, such as MeCP2, to the KRAB domain for better suppression efficacy [43]. Nonetheless, based on the outcomes from the reported system, we can flexibly modify the current toolbox by introducing additional transcriptional repressors or epigenetic modulators for robust suppression of any gene or gene network. The proof of concept generated here strongly encourages studies of different signaling pathways involved in sustaining malignant growth in melanoma cells. 

## Data Availability

The arrayed data have been provided as Supplementary Information.

## Supplementary Materials

The following supporting information can be downloaded at: https://www.mdpi.com/article/10.3390/cells12050730/s1, Figure S1: *S100B* expression, relative to *GAPDH*, was determined by quantitative real time PCR in B16-F1 and B16-F10 melanoma cell lines. Cells individually transduced with *S100B*-specific murine single guide RNAs (m-sgRNAs) did not show significant (*p* > 0.05) changes in gene expression, compared to parental cells (no transduction; negative control).; Figure S2: Significant increases in mRNA levels of *CDKN1A* (p21), but not *TP53* (p53) were observed in B16-F1 melanoma cells after transduction with dCas9-KRAB in combination with m-sgRNA-1, compared to control cells (no transduction or transduction with only dCas9-KRAB). In contrast, no significant changes in mRNA expression for both gene were observed in B16-F10 cells; Figure S3: B16-F10 melanoma cells transduced with dCas9-KRAB, with or without m-sgRNA-1, showed no noticeable apoptotic changes, such that apoptotic protein levels remained unaltered or were not cleaved; Table S1: *S100B* expression in PanCancer patient samples, as available in TCGA PanCancer Atlas Studies; Table S2: Age, gender and disease stage of the skin melanoma patients (*n* = 287), as available from the Skin Cutaneous Melanoma (TCGA, Firehose Legacy) study; Table S3: *S100B* expression, copy number alterations (CNA) and methylation values in skin cutaneous melanoma patients (*n* = 287), as available from the Skin Cutaneous Melanoma (TCGA, Firehose Legacy) study; Table S4: S100B protein expression in a cohort of melanoma patients (*n* = 23), who received immune checkpoint inhibitors (ICI), and their demographic and clinicopathological information; Table S5: Design of murine *S100B* specific sgRNA sequences, as used in the present study; Table S6: Primers used in the present study for PCR amplification of gblocks and sequencing analyses; Table S7: Primers used in the present study for PCR amplification of the target genes; Table S8: List of antibodies, used in performing Western Blotting.
