# Supplementary material for: CRISPR/dCas9-KRAB-Mediated Suppression of S100b Restores p53-Mediated Apoptosis in Melanoma Cells
Source: Cells. 2023 Feb 24;12(5):730. doi: 10.3390/cells12050730 (PMC10000373; doi:10.3390/cells12050730)
Supplement: Supplementary file 1 [file cells-12-00730-s001.zip › Supplementary Figures.pdf]

Supplementary Information

CRISPR/dCas9-KRAB-mediated suppression of *S100b* restores p53-mediated apoptosis in melanoma cells

Samrat Roy Choudhury[1*], Billie Heflin[2], Erin Taylor[2], Brian Koss[2], Nathan L Avaritt[2], Alan J Tackett[2*]

[1]Department of Pediatrics, Arkansas Children's Research Institute, University of Arkansas for Medical Sciences, Little Rock, AR 72202, USA. [2]Department of Biochemistry & Molecular Biology, University of Arkansas for Medical Sciences, Little Rock, AR, USA.

Corresponding Author

Alan J Tackett

Department of Biochemistry & Molecular Biology, University of Arkansas for Medical Sciences, Little Rock, AR 72205, USA, Email: AJTackett@uams.edu, Tel: +1 (501) 686-8152.

ORCID: 0000-0002-3672-4460

Samrat Roy Choudhury, Pediatric Hematology-Oncology, Arkansas Children's Research Institute, University of Arkansas for Medical Sciences, Little Rock, AR 72202, USA. Email: sroychoudhury@uams.edu, Tel: +1 (501) 364-7531.

ORCID: 0000-0002-6555-3031

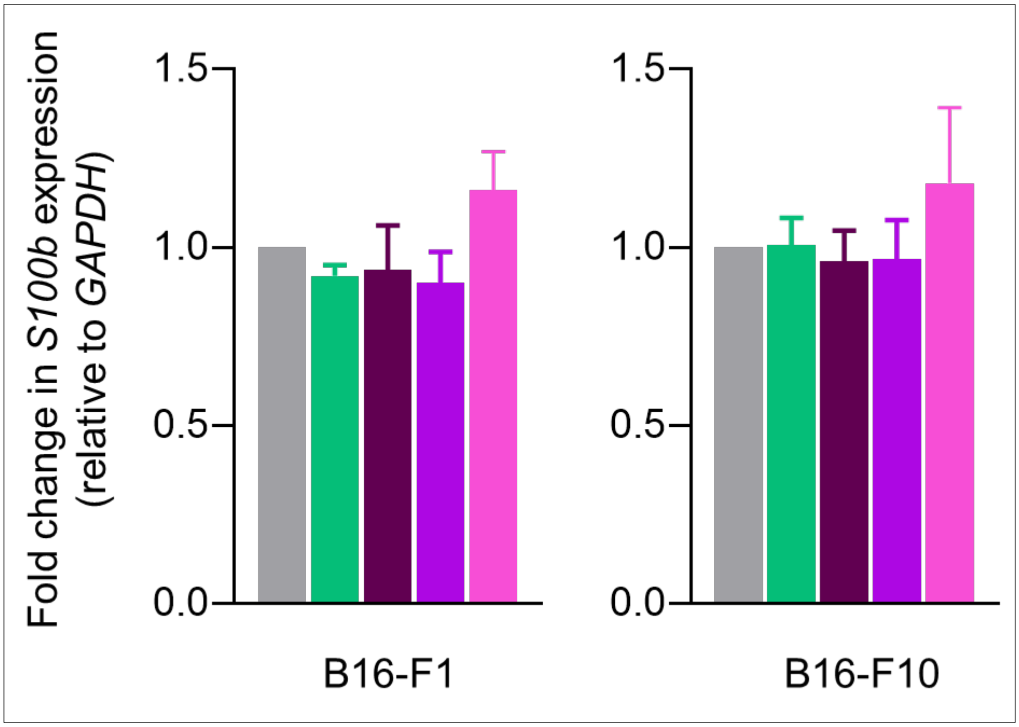

Fig. S1. *S100B* expression, relative to *GAPDH*, was determined by quantitative real time PCR in B16-F1 and B16-F10 melanoma cell lines. Cells individually transduced with S100B-specific murine single guide RNAs (m-sgRNAs) or EGFP did not show significant ($p < 0.05$) changes in gene expression, compared to parental cells (no transduction; negative control).

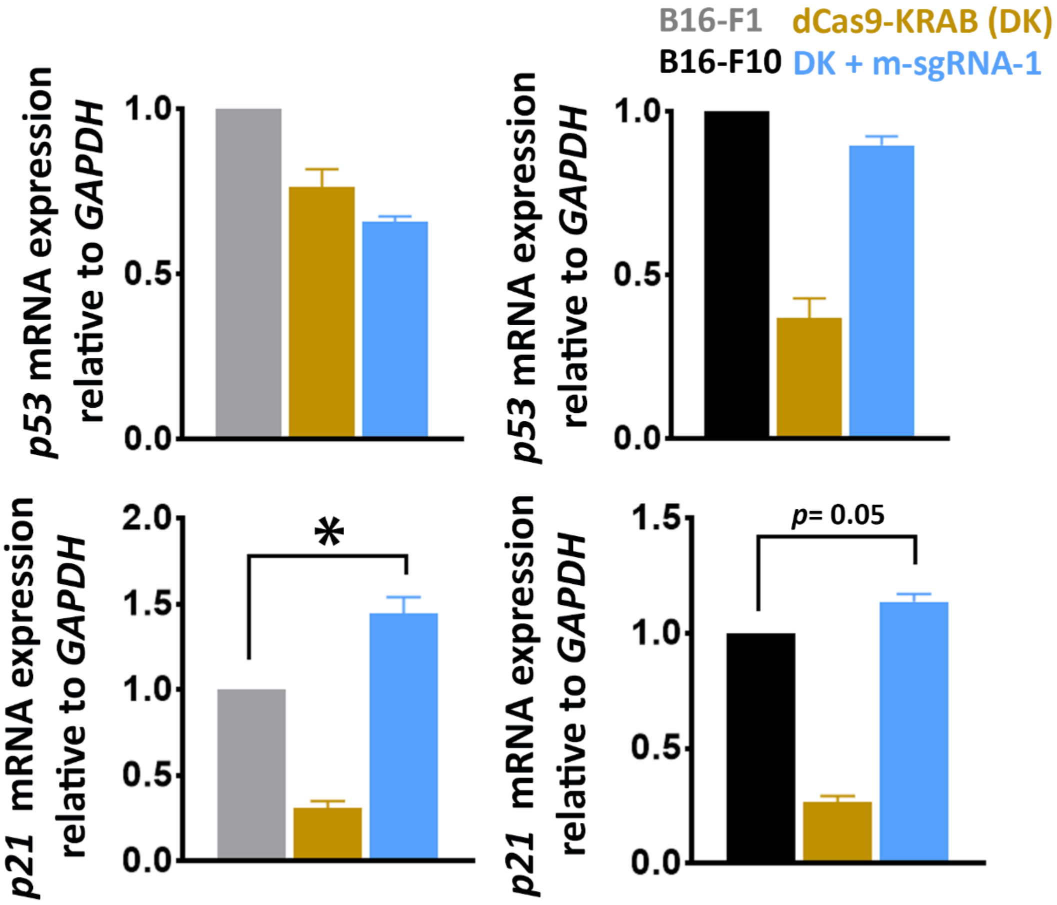

Fig. S2. Significant increases in mRNA levels of *CDKN1A* (p21), but not *TP53* (p53) were observed in B16-F1 melanoma cells after transduction with dCas9-KRAB in combination with m-sgRNA-1, compared to control cells (no transduction or transduction with only dCas9-KRAB). In contrast, no significant changes in mRNA expression for both gene were observed in B16-F10 cells.

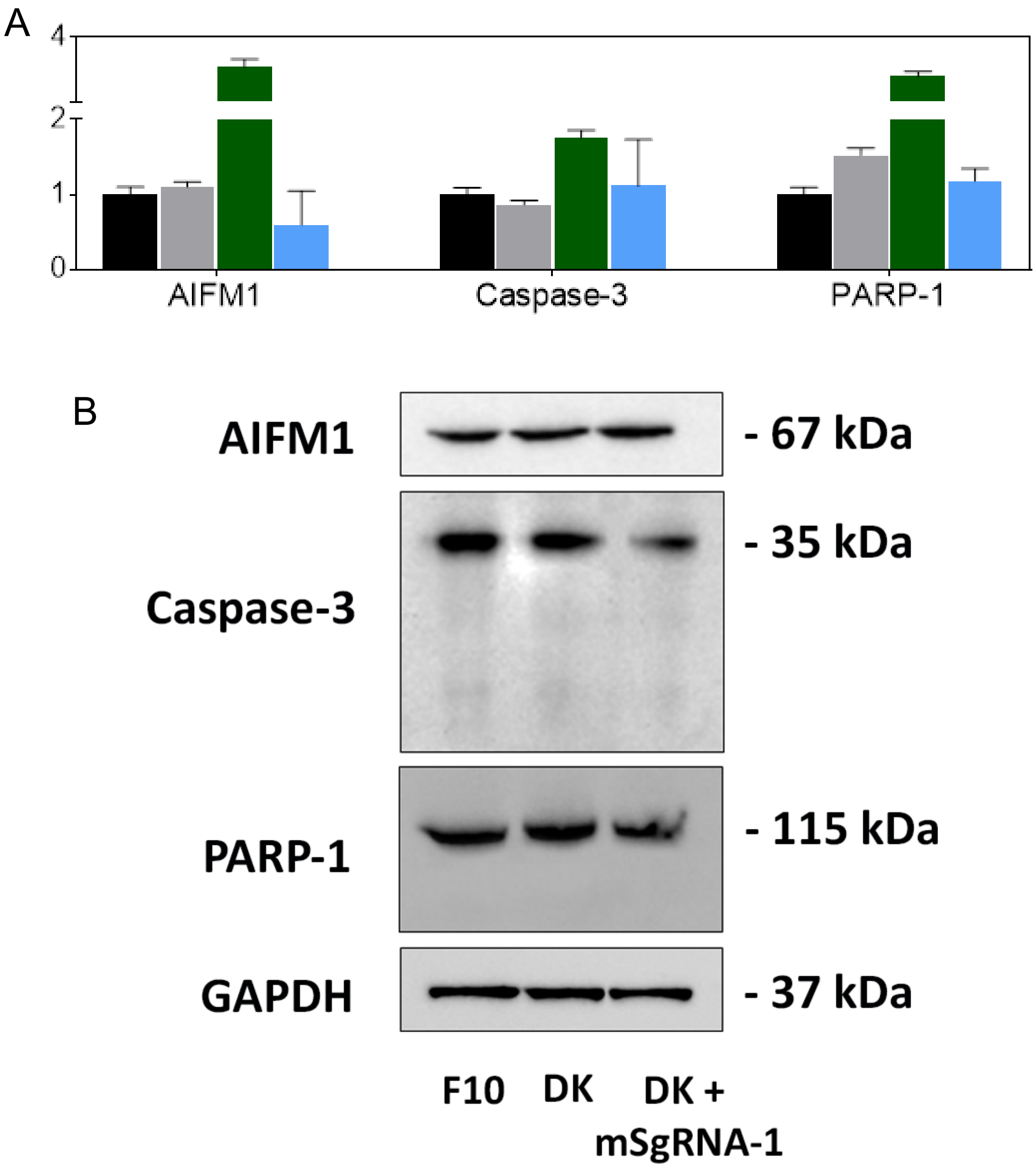

Fig. S3. (A) The mRNA expression of apoptosis related genes such as AIFM1, Caspase-3, or PARP-1 did not increase significantly ($p<0.05$) upon transduction with dCas9-KRAB alone, or in combination with sgRNAs, specific to S100b (m-sgRNA-1), compared to the untreated B16-F10 cells. In contrast, the combination of dCas9+KRAB and sgRNA specific to EGFP resulted in an unexplained anomalous increase in apoptotic gene expression. **(B)** Protein expression of the same apoptotic genes remained unaltered, whereas no cleaved fraction of caspase-3 was

formed, when the B16-F10 cells were transduced with dCas9-KRAB alone or in combination with S100b specific m-sgRNA-1.
